# Investigating Machine Learning and Control Theory Approaches for Process Fault Detection: A Comparative Study of KPCA and the Observer-Based Method

**DOI:** 10.3390/s23156899

**Published:** 2023-08-03

**Authors:** Fatma Lajmi, Lotfi Mhamdi, Wiem Abdelbaki, Hedi Dhouibi, Khaled Younes

**Affiliations:** 1 National Engineering School of Sousse, ENISO Laboratory: Networked Objects, Control, and Communication Systems (NOCCS), Sousse 4054, Tunisia; 2 National School of Engineering Monastir, Rue Ibn ELJazzar, Monastir 5019, Tunisia; lotfienim@yahoo.fr; 3 College of Engineering and Technology, American University of the Middle East, Egaila 54200, Kuwait; khaled.younes@aum.edu.kw; 4 High Institute of Applied Sciences and Technology of Kairouan, University of Kairouan, Kairouan 3100, Tunisia; hedi.dhouibi@laposte.net

**Keywords:** KPCA, observer-based method, machine learning, unsupervised learning, nonlinear process monitoring, fault detection

## Abstract

The paper focuses on the importance of prompt and efficient process fault detection in contemporary manufacturing industries, where product quality and safety protocols are critical. The study compares the efficiencies of two techniques for process fault detection: Kernel Principal Component Analysis (KPCA) and the observer method. Both techniques are applied to observe water volume variation within a hydraulic system comprising three tanks. PCA is an unsupervised learning technique used for dimensionality reduction and pattern recognition. It is an extension of Principal Component Analysis (PCA) that utilizes kernel functions to transform data into higher-dimensional spaces, where it becomes easier to separate classes or identify patterns. In this paper, KPCA is applied to detect faults in the hydraulic system by analyzing the variation in water volume. The observer method originates from control theory and is utilized to estimate the internal states of a system based on its output measurements. It is commonly used in control systems to estimate the unmeasurable or hidden states of a system, which is crucial for ensuring proper control and fault detection. In this study, the observer method is applied to the hydraulic system to estimate the water volume variations within the three tanks. The paper presents a comparative study of these two techniques applied to the hydraulic system. The results show that both KPCA and the observer method perform similarly in detecting faults within the system. This similarity in performance highlights the efficacy of these techniques and their potential adaptability in various fault diagnosis scenarios within modern manufacturing processes.

## 1. Introduction

The contemporary manufacturing landscape necessitates heightened product quality and safety operational practices. To maintain optimal system functionality and reduce downtime in case of failure, the early detection of process faults is critical [1]. Consequently, several process monitoring-based Multivariate Statistical Process (MSP) methods were developed thanks to their efficiencies and simplicity [2,3].

The Kernel Principal Component Analysis (KPCA) method, a simple yet interesting technique developed by Ratch et al. [4], is designed to accurately model nonlinear relationships inherent in process data. Utilizing the principle of kernel tricks [5], KPCA can efficiently project the input data with linearly inseparable structures onto a higher dimensional feature space in which the data become linearly separable, facilitating the execution of conventional Principal Component Analysis (PCA) within the feature space. KPCA has shown good results in analysis, modeling, and fault detection accuracy across a range of applications such as face recognition [6], speech recognition, nonlinear process monitoring, and fault diagnosis [7,8].

The observer-based method is commonly employed in Fault Detection (FD). The fundamental concept behind the observer or filter-based technique involves approximating the system’s states using the collected measurement data. Consequently, the estimated states are compared to the measured states of the monitored system to produce the residual.

Different attempts have addressed the incorporation of the observer in control systems, specifically in the domains of electrical drives [9,10] and robotics [11]. The primary objective when designing the observer for control applications should focus on accurate state estimation.

The estimation of unmeasured states differs from the design of observers for fault detection, which focuses on estimating measured states [12]. Numerous studies have explored observer-based FD in various contexts [13,14,15].

For instance, Khalid et al. [16] detected sensor faults in small autonomous helicopters using Observer/Kalman filter identification. Yang and Liu [17] employed a Kalman filter for fault diagnosis and a hybrid genetic adaptive neuro-fuzzy inference system for fault classification.

Alkaya and Eker [18] introduced a state estimation approach using Kalman filtering to predict failures and prevent maintenance in a DC motor. Tarantino et al. [19] detected sensor faults in DC and BLDC motors using Luenberger observers.

Several issues are emerging, mainly related to fault diagnosis. The majority of them investigate single faults. However, a more intricate issue demands attention, namely the detection and localization of multiple faults-a situation where multiple breakdowns occur and overlap in time. We assess the system using a fault injection approach, where we deliberately introduce faults and observe system responses while adjusting the time parameter each time.

This problem occurs in many industrial systems. Nonetheless, the research dedicated to this domain remains limited due to the complexity of the task and the combinatorial explosion problem. In fact, the complexity of the current systems, with their multiple functionalities, makes them susceptible to multiple faults, which need to be considered simultaneously. The heterogeneity of the data that should be explored for diagnosis poses an additional challenge and requires adapted methods. The purpose of this paper is to propose a method for fault diagnosis in complex physical systems. Techniques derived from machine learning and control theory are used for this purpose, KPCA [20] and the observer method [21,22], respectively. We used KPCA and observer methods to simulate and detect faults in the three-tank system.

The rest of the article is structured as follows: Section 2 presents KPCA and its fault detection index. Section 3 introduces the observer approach and its underlying principles. In Section 4, a comparative study between the observer approach and the kernel method for fault detection in a hydraulic system with three tanks is presented. Conclusions are delivered at the conclusion of the paper.

## 2. Kernel Principal Component Analysis (KPCA)

Kernel Principal Component Analysis (KPCA) is a nonlinear extension of the traditional Principal Component Analysis (PCA) technique. PCA is a widely used method for dimensionality reduction and feature extraction in data analysis. However, PCA assumes linearity in the data, which limits its applicability to linearly separable or well-behaved datasets.

KPCA overcomes this limitation by employing a kernel function to map the input data into a higher-dimensional feature space, where it becomes easier to find nonlinear relationships and patterns. In this feature space, KPCA applies PCA to extract the principal components, which are the directions of maximum variance in the data.

The kernel function in KPCA allows for implicit computations in the high-dimensional space without explicitly transforming the data. Commonly used kernel functions include the Gaussian (or radial basis function), polynomial, and sigmoid kernels. These kernels measure the similarity or dissimilarity between data points, enabling the extraction of nonlinear features that would be challenging to capture using linear techniques.

The KPCA algorithm involves three main steps: (1) computation of the kernel matrix, which stores the pairwise similarities between data points based on the chosen kernel function, (2) agenda composition of the kernel matrix to obtain the principal components, and (3) projection of the data onto the principal components to obtain the transformed features.

KPCA finds applications in various fields, including computer vision, pattern recognition, bioinformatics, and signal processing. It enables the detection of nonlinear patterns, clustering of complex data, and nonlinear dimensionality reduction. By leveraging the power of nonlinear mapping, KPCA offers a valuable tool for analyzing and extracting meaningful information from high-dimensional and nonlinear datasets for education and analysis [23,24]. It consists of transforming the nonlinear aspects of input data space E into linear ones within a newly high-dimensional feature space, denoted H, and to perform PCA in that space. The feature space H is nonlinearly transformed from the input space E with a non-linear mapping function ϕ. The mapping of sample x∈E in the feature space H can be written as:

(1) $$\begin{array}{c} \phi :E\;\subset R^{m}\to H\subset R^{H}\\ x\to \phi (x) \end{array}$$

Let us consider $X={\left[x_{1},\ldots ,x_{i},\ldots ,x_{N}\right]}^{T}$ the training data matrix scaled to zero mean and unit variance. Where $x_{i}\in E\subset R^{m}$ is a data vector, N is the number of observation samples and m is the number of process variables.

The monitoring phase based on the linear PCA approach requires the selection of principal components that maximize the variance in the data set. This is accomplished using the eigen decomposition of the covariance matrix. Similarly, this approach was generalized in the Kernel PCA approach by Ratsch [4]. The covariance matrix $C_{\Phi }$ in the feature space H is given by:

(2) $$C_{\Phi }=\frac{1}{N}\sum_{i=1}^{N}\phi (x_{i})\phi (x_{i}{)}^{T}$$

Let $\chi ={\left[\phi (x_{1})\ldots \phi (x_{i})\ldots \;\phi (x_{N})\right]}^{T}\in R^{N\times h}$ define the data matrix in the feature space H, then $C_{\Phi }$ can be expressed as:

(3) $$C_{\Phi }=\frac{1}{N}\chi^{T}\chi$$

The principal components of the mapped data $\phi (x_{1})\ldots \phi (x_{i})\ldots \;\phi (x_{N})$ are computed by solving the eigenvalue decomposition of $C_{\Phi }$, such that:

(4) $$\lambda_{j}\mu_{j}=C_{\Phi }\mu_{j}\;\mathrm{with}\;j=1,\ldots ,h$$

With $\mu_{j}$ being the jth eigenvector and $\lambda_{j}$ the associated jth eigenvalue. For $\lambda_{j}\neq 0$, there exist coefficients $\alpha_{i,j};i=1\ldots N$, such that all eigenvectors $\mu_{j}$ can be considered as a linear combination of $\left[\phi (x_{1})\;\phi (x_{2})\ldots \;\phi (x_{N})\right]$ and can be expressed by:

(5) $$\mu_{j}=\sum_{i=1}^{N}\alpha_{i,j}\phi (x_{i})$$

However, in practice, the mapping function ϕ is not defined and then the covariance matrix $C_{\Phi }$ in the feature space cannot be calculated implicitly. Thus, instead of solving the eigenvalue problem directly on $C_{\Phi }$, we apply the kernel trick firstly used for Support Vector Machine (SVM) [25]. The inner product given in Equation (2) may be calculated by a kernel function k that satisfies Mercer’s theorem [12] as follows:

(6) $${\left\langle \phi (x),\phi (x^{\prime })\right\rangle }_{H}=k(x,x^{\prime })\forall x,x^{\prime }\in R^{m}$$

Let us define a kernel matrix K associated with a kernel function k as:

(7) $$K=\chi \chi^{T}=\left[\begin{array}{ccc} k(x_{1},x_{1}) & \cdots & k(x_{1},x_{N})\\ \vdots & \ddots & \vdots \\ k(x_{N},x_{1}) & \cdots & k(x_{N},x_{N}) \end{array}\right]\in R^{N\times N}$$

Applying the kernel matrix may reduce the problem of the eigenvalue decomposition of $C_{\Phi }$ [26]. Hence, eigendecomposition of the kernel matrix K is equivalent to performing PCA in $R^{H}$, so that:

(8a) sNΛV=KV

where Λ is the diagonal matrix of eigenvalues $\lambda_{j}$ arranged in descending order

(8b) $$\Lambda =diag(\lambda_{1}\ldots \lambda_{j}\ldots \lambda_{N})$$

and V is the matrix of their corresponding eigenvectors.

(8c) $$V=\left[\alpha_{1}\ldots \alpha_{j}\ldots \alpha_{N}\right]$$

Since the principal components are orthonormal, it is required to guarantee the normality of $\mu_{j}$ in Equation (4), such that:

(9) $${\left\langle \mu_{j},\mu_{j}\right\rangle }_{H}=1;j=1\ldots n$$

n is the number of the first non-zero eigenvalues.

### 2.1. Number of Principal Components

Determining the number of retained principal components (l) is an important step of modeling based on KPCA. The Cumulative Percent Variance (CPV) has been proposed to compute the retained PC (l) [27,28]. The cumulative percent variance (CPV) is the sum of the first l eigenvalues divided by their total variations. It can be expressed as:

(10) $$CPV(l)=\frac{\sum_{j=1}^{l}\lambda_{j}}{\sum_{j=1}^{m}\lambda_{j}}100\%$$

The number l of retained PCs is chosen if the CPV is higher than 95%.

### 2.2. Fault Detection

Like in the PCA approach, the squared prediction error (SPE) is usually used for fault detection using KPCA [29,30]. However, the conventional KPCA does not provide any approach to data reconstruction in the feature space. Thus, the computation SPE index is difficult in the KPCA method. Kim [31] and Lahdhiri [32] proposed a simple expression to calculate SPE in the feature space H, which is shown as follows:

(11) $$SPE=\left\|k(x,x)-k_{x_{t}}^{T}\hat{P}{\hat{\Lambda }}^{-1}{\hat{P}}^{T}k_{x_{t}}\right\|$$

where $\hat{P}=\left[\alpha_{1},\ldots ,\alpha_{l}\right]$ is the matrix of the first l principal eigenvectors of K, $\hat{\Lambda }=diag\left[\lambda_{1},\ldots ,\lambda_{l}\right]$ is the diagonal matrix of the first l eigenvalues of K [33], and $k_{x_{t}}={\left[k(x_{1},x_{t}),\ldots ,k(x_{N},x_{i}),\ldots ,k(x_{N},x_{t})\right]}^{T};i=1\ldots N$.

The confidence limit for the SPE index can be calculated using the $\chi^{2}$ distribution and is given by:

(12) $${SPE}_{lim}\approx \delta_{\alpha }^{2}$$

where $\delta_{\alpha }^{2}$ is the control limit expressed by:

(13) $$\delta_{\alpha }^{2}=g\chi_{h,\alpha }^{2}$$

with: $g=\frac{b}{2a}$ and $h=\frac{2a^{2}}{b}$, where a is the estimated mean and b is the variance of the SPE [34,35].

## 3. The Observer Method

In fault diagnosis, an observer is a mathematical model or algorithm used to estimate the state variables and fault parameters of a system based on available measurements. The structure of an observer varies depending on the type of system being observed and the nature of the faults being diagnosed. However, the general structure of an observer typically involves the following components:

- System Model: The observer relies on a mathematical model that describes the dynamics of the system being observed. This model can be derived from first principles or obtained through system identification techniques.
- Measurement Equation: The observer uses a measurement equation that relates the system’s state variables to the available measurements. This equation can be derived from the system model and typically includes sensor equations and/or sensor noise models.
- State Estimation: The core of the observer is the state estimation algorithm, which updates and estimates the system’s state variables based on the available measurements. Various estimation techniques can be used, such as Kalman filters, extended Kalman filters, particle filters, or model-based observers like the sliding mode observer.
- Fault Detection: In fault diagnosis, the observer is also responsible for detecting the occurrence of faults. This can be done by comparing the estimated state variables with expected values or by analyzing the residuals between the measurements and the estimated values.
- Fault Parameter Estimation: If faults are detected, the observer may also estimate the fault parameters, such as fault magnitudes, locations, or characteristics. This is typically done by incorporating fault models into the observer and updating the estimated parameters based on the available measurements and fault detection results.
- Adaptation and Learning: Depending on the observer’s design, it may incorporate adaptation or learning mechanisms to improve its performance over time. These mechanisms allow the observer to adapt to changes in system dynamics or fault characteristics, or to learn from historical data to enhance its fault diagnosis capabilities.

It is important to note that the specific structure and algorithm used for fault diagnosis observers can vary greatly depending on the application, system complexity, and available information. Different domains, such as power systems, automotive, or aerospace, may have specialized observer designs tailored to their specific requirements. A system with *p* inputs denoted *u(t)* and m output measurements denoted *x(t)*.

The dynamic behavior of this system is described by the following equations [36]:

(14) $$\dot{x}(t)=Ax(t)+Bu(t)$$

(15) $$\begin{array}{c} y(t)=Cx(t)\\ x(0)=x_{0} \end{array}$$

where $x(t)\in R^{n}$ is the state vector, Ref. [37] is the output vector, and $u(t)\in R^{r}$ is the input vector.

Note that matrices *A*, *B*, and *C* represent the state-space description of a linear time-invariant system [38,39], and they have appropriate dimensions with those of the vectors x(t), $u\left(t\right),$ and y(t).

Given that the state is not generally available [40], the objective is an observer in order to perform a feedback control condition and estimate this state by a variable which we denote as $\hat{x}(t)$. This estimate is carried out by a dynamic system, the output will be precisely $\hat{x}\left(t\right),$ and the input will consist of all the information available [41], that is to say, *u(t)* and *y(t).* The structure of an observer can be written as:

(16) $$\begin{array}{c} \dot{\hat{x}}(t)=A\hat{x}(t)+Bu(t)+K(y(t)-\hat{y}(t))\\ \hat{y}(t)=C\hat{x}(t) \end{array}$$

where the correction term appears clearly in terms of the reconstruction error of the output $y(t)-\hat{y}(t)$, and the correct term can be written as an ion gain [42], L, or the determined observer gain [43]. This structure can be written as:

(17) $$\dot{\hat{x}}(t)=(A-KC)\hat{x}(t)+Bu(t)+Ky(t)$$

If we consider the estimation error: $\tilde{x}(t)=x(t)-\hat{x}(t)$, we obtain: $\dot{\tilde{x}}(t)=(A-KC)\tilde{x}(t)$.

**Figure 1:**
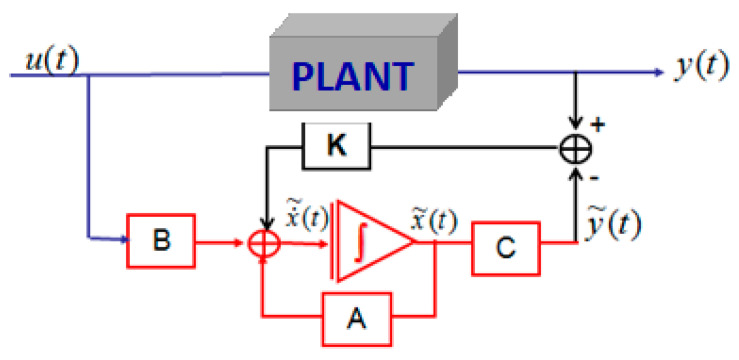
Structure of the observer.

The observer described by Equation (15) is illustrated in Figure 1.

### Fault Detection Observer

In this work, we assume that the actuators and sensors are affected by faults. Our goal is to detect and isolate the faults [43]. The state of the system model can be written as:

(18) $$\begin{array}{c} \dot{x}(t)=Ax(t)+B(u(t)+f_{a}(t))\\ y(t)=Cx(t)+f_{c}(t) \end{array}$$

where $f_{a}(t)$ is the actuator fault of the actuators and ${\;f}_{c}(t)$ is a sensor fault.

(19) $$\begin{array}{c} \dot{\hat{x}}(t)=(A-KC)\hat{x}(t)+Bu(t)+Ky(t)\\ \hat{y}(t)=C\hat{x}(t) \end{array}$$

where $\hat{x}(t)\in R^{n}$ is an estimated state vector, K is a matrix such that *A* − *KC* is stable and its proper values have a real part smaller than A. This leads to:

(20) $$\begin{array}{c} \dot{\tilde{x}}\left(t\right)=\left(A-KC\right)\tilde{x}\left(t\right)+Bf_{a}\left(t\right)-Kf_{c}\left(t\right)\\ \tilde{y}(t)=C\tilde{x}(t)+f_{c}(t) \end{array}$$

If K is such that A−KC is a Hurwitz matrix, the residue $\tilde{y}(t)$ tends to 0 well in the absence of defects. Transfer between faults and residuals can be written:

(21) $$\tilde{y}(t)=C(pI-A+KC{)}^{-1}Bf_{a}(t)+\left[I-C(pI-A+KC{)}^{-1}K\right]f_{c}(t)$$

where *p* is a temporal derivative operator.

Which leads to [44], taking into account the inversion lemma in:

(22) $$(A+BCD{)}^{-1}=A^{-1}-A^{-1}B(C^{-1}+DC^{-1}B{)}^{-1}DA^{-1}$$

which can be written as:

(23) $$\left[I+C(pI-A{)}^{-1}K\right]\tilde{y}(t)=C(pI-A{)}^{-1}Bf_{a}(t)+f_{c}(t)$$

From this relationship and in the absence of actuator failure, the system for isolating fault sensors from residues can be written as:

(24) $$\dot{\eta }(t)=A\eta (t)+K\tilde{y}(t)$$

(25) $$f_{a}(t)=C\eta (t)+\tilde{y}(t)$$

This variable is estimated using the same system as before, that is to say:

(26) $$\begin{array}{c} \dot{\eta }(t)=A\eta (t)+K\tilde{y}(t)\\ \xi (t)=C\eta (t)+\tilde{y}(t) \end{array}$$

And the estimation of sensor failure is given by the inversion of the initial model of the system:

(27) $$\beta (t)=\Delta (p){\left[C(pI-A{)}^{-1}B\right]}^{-1}\xi (t)$$

where $\Delta (p)=diag\left\{\frac{1}{(p+\alpha {)}^{i}}\right\}$ is the matrix filters as $\Delta (p){\left[C(pI-A{)}^{-1}B\right]}^{-1}$ is bi-causal [45].

β(t) is not an estimate of faults but rather filtering defects; however, the character of diagonal Δ(p) allows the isolation [20].

## 4. Applications

### 4.1. Overview of Three-Tank System Applications

A three-tank water system, also referred to as a triple-tank or multi-tank system, exhibits a water storage and distribution network, using multiple tanks for various functions. Specific uses for three-tank water systems vary, but typically fall within environmental engineering, including Rainwater Harvesting and Reuse systems, greywater recycling, and off-grid water supply [46,47,48]. These applications have found their place in the heart of targeting sustainable practices, more specifically SDG6, by ensuring Clean Water and Sanitation [46]. A three-tank system can be employed for rainwater harvesting and reuse, with each tank serving its own specific function. One tank could collect rainwater from the roof, another could be dedicated to filtering and treating (primary, secondary, and tertiary water treatment processes), while a third could store treated rainwater for urban reuse in fields such as irrigation. This system helps decrease the reliance on freshwater sources while supporting sustainable water management practices [46].

Triple tank systems provide an effective method of greywater recycling via treating and reusing urban residual water, generated from domestic activities like bathing, handwashing, and laundry. One tank collects urban water for disinfection processes (mostly chlorine and combined chlorine sub-products) [49], while a second tank stores treated water for reuse, in activities such as toilet flushing or landscape irrigation. This approach helps release the stress on freshwater resources while alleviating strain on sewerage systems [47].

In remote or off-grid locations, the availability of reliable water resources is limited. Hence, a three-tank water system can serve as a reliable self-sufficient water supply source. One tank could hold water collected from natural sources like wells or springs, while another would treat and purify it before the third would store the treated water for domestic consumption [48].

On the other hand, several shortcomings and limitations may arise from the installation of three-tank systems. Following the sub-division of the system into three counterparts, the urge for complexity and space requirements are more demanding [46,47,48]. Additional space must also be implemented, as well as the multiple features required to connect the tanks (pumps, valves, etc.). In addition, this system exhibits low cost-effectiveness, as it involves high installation prices compared to the simple water storage system [50]. The maintenance and additional unit operations, required for water screening, treatment, and distribution, will incur additional expenses [51]. Following the complexity of the system, more design heuristics and trade-offs should be considered. This will make the maintenance operation more tedious and time-consuming [51]. On the human factor and engineering intuition level, a high level of skill and knowledge of handling are required, for the sake of preserving the integrity of the system [46,47,48].

Most of the aforementioned factors could be overcome by implementing a proper control system, based on the fact that multiple error sources could be generated. KPCA exhibits a highly convenient unsupervised machine learning approach for a three-tank system control, as it involves dealing with intercorrelated data input [52]. In other words, the error in one part of the system will definitely influence the other components.

### 4.2. Process Description of a Hydraulic System with Three Tanks

**Figure 2:**
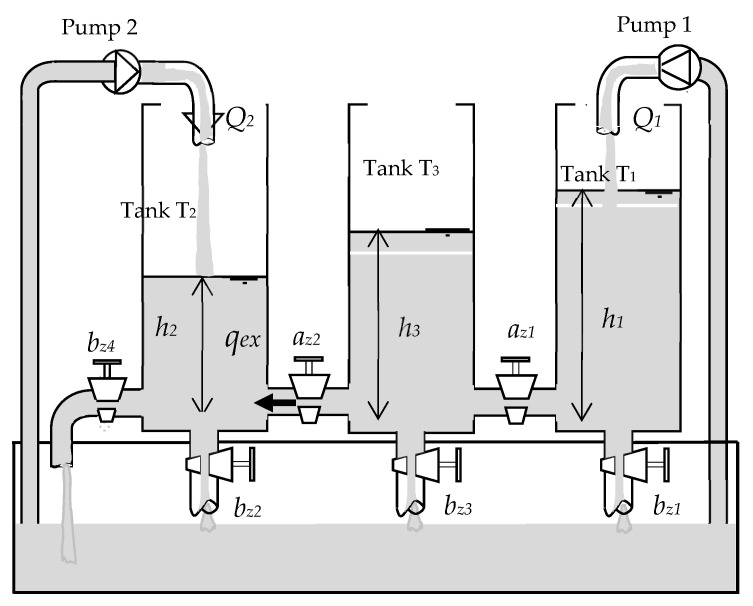
Three-tank system.

As illustrated in Figure 2, the considered process is a three-tank system with two inputs and three outputs. It consists of three tanks with identical sections, supplied with distilled water. They are serially interconnected by two cylindrical pipes with identical sections [53,54]. The pipes of communication between tanks T_1_ and T_2_ are equipped with manually adjustable valves; the flow rates of the connection pipes can be controlled using ball valves *a_z_*_1_ and *a_z_*_2_. The plant has one outlet pipe located at the bottom of tank T_3_. There are three other pipes each installed at the bottom of each tank, which are provided with a direct connection (outflow rate) to the reservoir with ball valves *b_z_*_1_, *b_z_*_2_, and *b_z_*_3_, respectively, The pipes can only be manipulated manually [12]. Pumps 1 and 2 are supplied by water from the reservoir with flow rates *Q*_1_*(t)* and *Q*_2_*(t)*, respectively. The necessary level measurements *h*_1_*(t)*, *h_2_(t)*, and *h*_3_*(t)* are carried out by the piezo-resistive differential pressure sensors.

The state equations are obtained by writing that the variation of the water volume in a tank is equal to the difference between the incoming flow and the outgoing flows which means the water of tanks 1 and 2 can flow toward tank 3.

Then, the system can be represented by the following equations:

(28) $${\dot{h}}_{i}(t)=\frac{1}{A}(Q_{i}^{in}(t)-Q_{ij}^{out1}(t)-Q_{ij}^{out2}(t))i,j=1,2,3$$

where $Q_{i}^{in}(t)$ is the flow through pump *i* (*i* = 1; 2), and $Q_{ij}^{out1}(t)$ represents the flow rates of water between tanks *i* and *j* (i,j=1,2,3∀i≠j), and can be expressed using the law of Torricelli [29].

(29) $$Q_{ij}^{out1}(t)=a_{zi}S_{n}sign(h_{i}-h_{j})\sqrt{2g\left|h_{i}-h_{j}\right|},j=1,3$$

and $Q_{ij}^{out2}(t)$ represents the outflow rate, given by:

(30) $$Q_{ij}^{out2}(t)=b_{zj}S_{L}\sqrt{2gh_{i}}\;j=1,\;2,\;3$$

where *hi(t)*, $Q_{i}^{in}\left(t\right),$ and $Q_{ij}^{out}(t)$ are, respectively, the levels of water, the input flow rate, and the output flow rate.

The parameters of the three-tank system are defined as follows. The controlled signals are the water levels (*h*_2_, *h*_3_) of tank 2 and tank 3. These levels are controlled by two pumps. The system can be considered as a multi-input multi-output system (MIMO) [54], where the input is inflow rates *Q*_1_ and *Q*_2_ and the output is liquid levels *h*_2_ and *h*_3_. Then the three-tank system can be modeled by the following three differential equations:

(31) $$\begin{array}{c} \frac{dh_{1}}{dt}=-c_{1}sign(h_{1}-h_{3})\sqrt{\left|h_{1}-h_{3}\right|}-B_{1}\sqrt{h_{1}}+\frac{Q_{1}}{a}\\ \frac{dh_{2}}{dt}=c_{3}sign(h_{3}-h_{2})\sqrt{\left|h_{3}-h_{2}\right|}-\left(B_{4}+B_{2}\right)\sqrt{h_{2}}+\frac{Q_{2}}{a}\\ \begin{array}{c} \frac{dh_{3}}{dt}=c_{1}sign(h_{1}-h_{3})\sqrt{\left|h_{1}-h_{3}\right|}-\left(B_{3}+B_{2}\right)\sqrt{h_{3}}-c_{3}sign(h_{3}-h_{2})\sqrt{\left|h_{3}-h_{2}\right|} \end{array} \end{array}$$

where the parameters *c_i_*, *i* = 1, 3 and *B_j_*, *j* = 1, 2, 3, 4 are defined by:

(32) $$c_{i}=\frac{1}{A}a_{zi}S_{n}\sqrt{2g}i=1,\;3$$

(33) $$B_{j}=\frac{1}{A}b_{zj}S_{L}\sqrt{2g}j=1,\;2,\;3,\;4$$

While taking *B*_1_ = *B*_2_ = *B*_3_ = 0, the three equations of the system become:

(34) $$\left\{\begin{array}{c} \frac{dh_{1}}{dt}=-c_{1}sign(h_{1}-h_{3})\sqrt{\left|h_{1}-h_{3}\right|}+\frac{Q_{1}}{a}\\ \frac{dh_{2}}{dt}=c_{3}sign(h_{3}-h_{2})\sqrt{\left|h_{3}-h_{2}\right|}-B_{4}\sqrt{h_{2}}+\frac{Q_{2}}{a}\\ \begin{array}{c} \frac{dh_{3}}{dt}=c_{1}sign(h_{1}-h_{3})\sqrt{\left|h_{1}-h_{3}\right|}-c_{3}sign(h_{3}-h_{2})\sqrt{\left|h_{3}-h_{2}\right|} \end{array} \end{array}\right.$$

At equilibrium, for a constant water level set point, the level derivatives must be zero.

(35) $${\dot{h}}_{1}={\dot{h}}_{2}={\dot{h}}_{3}=0$$

Therefore, using (31) in the steady state, the following algebraic relationship holds.

(36) $$\left\{\begin{array}{c} -c_{1}sign(h_{1}-h_{3})\sqrt{\left|h_{1}-h_{3}\right|}+\frac{Q_{1}}{a}=0\\ c_{1}sign(h_{1}-h_{3})\sqrt{\left|h_{1}-h_{3}\right|}-c_{3}sign(h_{3}-h_{2})\sqrt{\left|h_{3}-h_{2}\right|}=0 \end{array}\right.$$

For the coupled-tank system, the fluid flow *Q*_1_ into tank 1 cannot be negative because the pump can only drive water into the tank, then:

$$Q_{1}\geq 0$$

From (36), we have:

(37) $$c_{1}sign(h_{1}-h_{3})\sqrt{\left|h_{1}-h_{3}\right|}=\frac{Q_{1}}{a}$$

and $c_{1}sign(h_{1}-h_{3})\sqrt{\left|h_{1}-h_{3}\right|}=c_{3}sign(h_{3}-h_{2})\sqrt{\left|h_{3}-h_{2}\right|}$.

Then, (*h*_1_ − *h*_3_) ≥ 0 and (*h*_3_ − *h*_2_) ≥ 0. Therefore, if we assume

(38) $$x_{1}=h_{1},\;x_{2}=h_{2},\;x_{3}=h_{3},\;u_{1}=Q_{1}\;\mathrm{and}\;u_{2}=Q_{2}$$

We have

(39) $$\left\{\begin{array}{c} {\dot{x}}_{1}=-c_{1}\sqrt{x_{1}-x_{3}}+\frac{u_{1}}{a}\\ {\dot{x}}_{2}=c_{3}\sqrt{x_{3}-x_{2}}-B_{4}\sqrt{x_{2}}+\frac{u_{2}}{a}\\ {\dot{x}}_{3}=c_{1}\sqrt{x_{1}-x_{3}}-c_{3}\sqrt{x_{3}-x_{2}}\; \end{array}\right.$$

Which can be written as:

(40) $$\left\{\begin{array}{c} \dot{x}=f(t,x)+gu\\ y=cx \end{array}\right.$$

where

(41) $$x={\left[\begin{array}{ccc} x_{1} & x_{2} & x_{3} \end{array}\right]}^{T},\;u={\left[\begin{array}{cc} u_{1} & u_{2} \end{array}\right]}^{T},\;y={\left[\begin{array}{cc} x_{2} & x_{3} \end{array}\right]}^{T}$$

(42) $$f(t,x)=\left(\begin{array}{c} -c_{1}\sqrt{x_{1}-x_{3}}\\ c_{3}\sqrt{x_{3}-x_{2}}-B_{4}\sqrt{x_{2}}\\ c_{1}\sqrt{x_{1}-x_{3}}-c_{3}\sqrt{x_{3}-x_{2}} \end{array}\right),\;g=\left(\begin{array}{cc} \frac{1}{a} & 0\\ 0 & \frac{1}{a}\\ 0 & 0 \end{array}\right)\;\mathrm{and}\;c=\left(\begin{array}{ccc} 0 & 1 & 0\\ 0 & 0 & 1 \end{array}\right)$$

### 4.3. Simulation Results

We are interested in the fault detection of the pressure sensors, which measure the water levels (*h*_2_, *h*_3_) of tank 2 and tank 3 using conventional KPCA and the observer method. A total of 5000 samples were generated from this process [55]. The 1000 first samples were used to construct the KPCA model; the last 3000 samples are used to test the fault detection methods. We have used the Radial Basis Function (RBF) [56,57]. Two types of faults are considered [58]: faults in the pressure sensor of tank 2 and faults in the pressure sensor of tank 3.

Case 1: faults in the pressure sensor of tank 2 (water level *h*_2_).

Fault 1: a step bias of *h*_2_ by adding 10% more than its range of variation [59]. The fault is introduced between samples 1500 and 3000.

**Figure 3:**
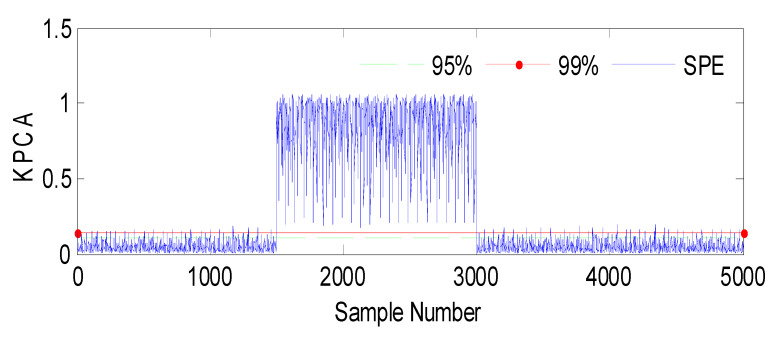
Evolution of the SPE index with Fault 1 affecting *h*_2_ using KPCA.

The SPE index is a statistical measure commonly used in multivariate analysis or process monitoring. It quantifies the discrepancy between predicted and observed values in a model. In this context, Figure 3 represents the results of an analysis or experiment involving a fault (Fault 1) and the SPE (Squared Prediction Error) index, with the variable *h*_2_ being affected. The evolution indicates that the figure shows changes or trends over time or some other continuous parameter. It suggests that the data or analysis captured a dynamic process or progression.

Fault 2: a step bias of *h*_2_ by adding 20% more than its range of variation. The fault is introduced between samples 3500 and 4500.

Case 2: Faults in the pressure sensor of tank 3 (water level *h*_3_)

Fault 1: a step bias of *h*_3_ by adding 10% more than its range of variation. The fault is introduced between samples 2000 and 3000 [60].

Fault 2: a step bias of *h*_3_ by adding 20% more than its range of variation. The fault is introduced between samples 3000 and 4000.

**Figure 4:**
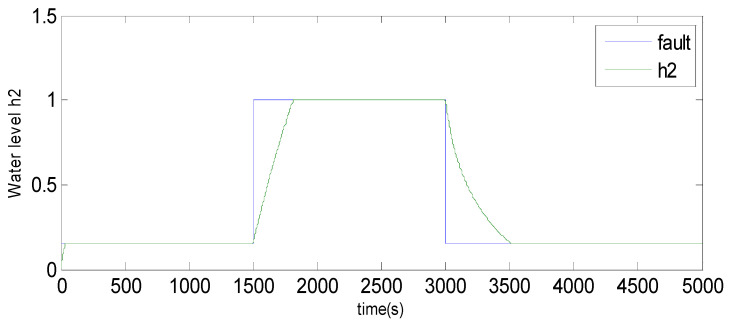
Evolution of sensor *h*_2_ between 1500 and 3000 using the observer method.

**Figure 5:**
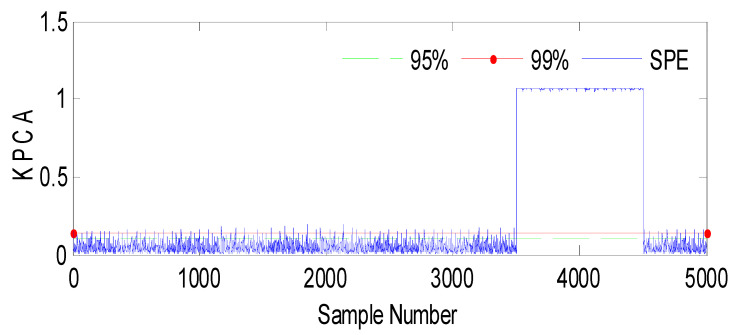
Evolution of the SPE index with Fault 2 affecting *h*_2_ using KPCA.

**Figure 6:**
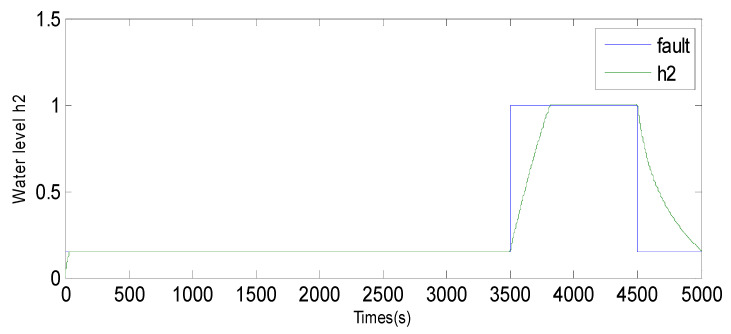
Evolution of sensor *h*_2_ between 3500 and 4500 using the observer method.

**Figure 7:**
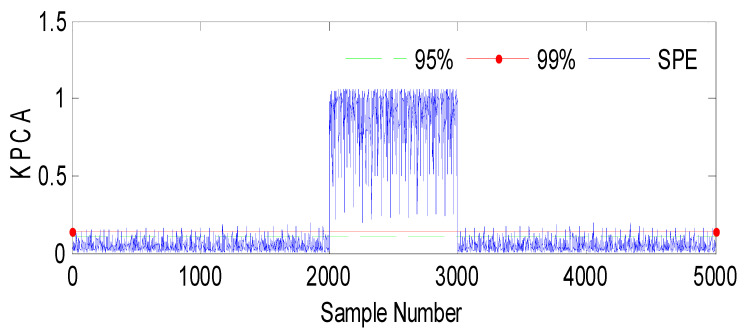
Evolution of the SPE index with Fault1 affecting *h*_3_ using KPCA.

**Figure 8:**
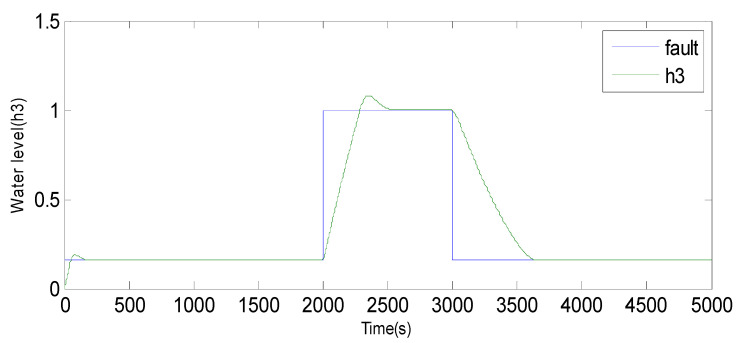
Evolution of sensor *h*_3_ between 2000 and 3000 using the observer method.

**Figure 9:**
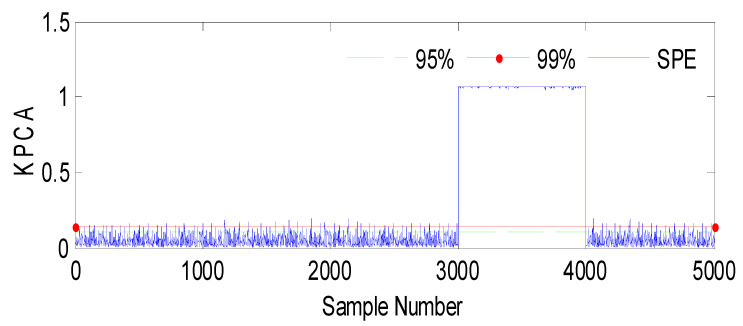
Evolution of the SPE index with Fault 2 affecting *h*_3_ using KPCA.

**Figure 10:**
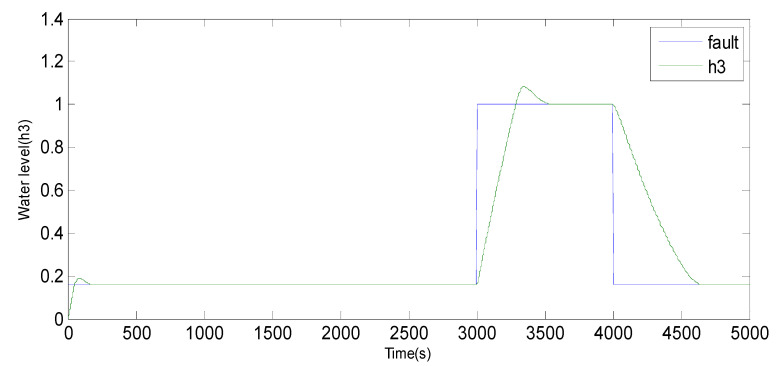
Evolution of sensor *h*_3_ between 3000 and 4000 using the observer method.

Based on the observations from Figure 4, Figure 5, Figure 6, Figure 7, Figure 8, Figure 9 and Figure 10, it is evident that both the kernel method and the observer technique detect a fault occurrence, as indicated by the defective outputs *h*_2_ and *h*_3_. Both methods yield effective and comparable results in terms of sensor fault detection. In conclusion, the simulation results using the “observer-based model and a kernel technique called KPCA” show that these two techniques are comparable in the field of fault diagnosis. These methods have been evaluated and demonstrated similar performances in terms of fault identification and detection. This suggests that the use of an observer-based model and the KPCA technique can be effective in diagnosing faults in a system. However, it is important to note that the comparison of performance between these techniques may depend on the specific context of the application and the characteristics of the system being studied. Further studies and experimental tests may be necessary to confirm these results and assess their applicability in other domains.

## 5. Conclusions

This paper offers a comprehensive examination of an observer-based model and a kernel technique called KPCA, both employed for sensor fault detection [61]. The observer’s operational principle is thoroughly explained, providing a detailed understanding of its function. To assess the effectiveness of these two techniques, a comparative analysis is conducted using a three-tank process. The simulation results yield valuable insights, demonstrating that both the observer-based model and the KPCA method yield satisfactory results. However, it is important to note that these findings are based on a single case study and cannot be considered in isolation. To establish the broader applicability of these techniques across various systems and conditions, further investigation is necessary. The technique used to validate our system is the injection of faults and we observe the reaction of the system with modification of the time each time. The way of identifying faults is validated and can be used in industry, specifically in chemical systems.

The problem is the difficulty of detecting defects. Our method has approved the capability of detecting defects despite the change in the nature of defects and the integration time.

Consequently, additional research is planned to advance the understanding of fault detection techniques through a deeper exploration of these approaches.

“Kernel Principal Component Analysis (KPCA) and observer-based approaches are two distinct methods commonly used in fault detection within the realms of machine learning and control theory. KPCA is a nonlinear dimensionality reduction technique that maps data into a high-dimensional feature space using kernel functions, enabling the detection of faults in complex and nonlinear systems. Its advantages lie in its ability to handle nonlinearity, making it suitable for intricate processes. However, KPCA’s performance heavily relies on the appropriate choice of kernel and its associated parameters, which can be challenging to determine in practice. On the other hand, observer-based approaches leverage mathematical models to estimate the system’s behavior and compare it with the actual response for detecting faults. The advantage of observer-based methods is their inherent robustness to disturbances and noise, allowing them to perform well in noisy environments. Nevertheless, these approaches often require a comprehensive and accurate model of the system, and their performance may suffer if the model is not precise or if the system exhibits significant nonlinear behavior. Overall, choosing between KPCA and an observer-based approach depends on the specific characteristics of the system, the availability of accurate models, and the level of nonlinearity present, as each method offers distinct strengths and weaknesses in fault detection applications”.

## Data Availability

Not applicable.

## References

[1] Lima A., Zen H., Nankaku Y., Tokuda K., Kitamura T., Resende F.G. (2005). Applying Sparse KPCA for Feature Extraction in Speech Recognition. IEICE Trans. Inf. Syst..

[2] Aronszajn N. (1950). Theory of Reproducing Kernels. Trans. Am. Math. Soc..

[3] Baklouti R., Mansouri M., Nounou M., Nounou H., Hamida A.B. (2016). Iterated Robust Kernel Fuzzy Principal Component Analysis and Application to Fault Detection. J. Comput. Sci..

[4] Ratsch G. (1999). Input Space versus Feature Space in Kernel-Based Methods. IEEE Trans. Neural Netw..

[5] Botre C., Mansouri M., Nounou M., Nounou H., Karim M.N. (2016). Kernel PLS-Based GLRT Method for Fault Detection of Chemical Processes. J. Loss Prev. Process Ind..

[6] Chetouani Y. (2008). A Neural Network Approach for the Real-Time Detection of Faults. Stoch. Environ. Res. Risk Assess..

[7] Cho J.-H., Lee J.-M., Choi S.W., Lee D., Lee I.-B. (2005). Fault Identification for Process Monitoring Using Kernel Principal Component Analysis. Chem. Eng. Sci..

[8] Choi S.W., Lee C., Lee J.-M., Park J.H., Lee I.-B. (2005). Fault Detection and Identification of Nonlinear Processes Based on Kernel PCA. Chemom. Intell. Lab. Syst..

[9] Doan P.T., Bui T.L., Kim H.K., Kim S.B. Sliding-Mode Observer Design for Sensorless Vector Control of AC Induction Motor. Proceedings of the 2013 9th Asian Control Conference (ASCC).

[10] Ruderman M., Iwasaki M. Sensorless Control of Motor Velocity in Two-Mass Actuator Systems with Load Sensing Using Extended State Observer. Proceedings of the 2014 IEEE/ASME International Conference on Advanced Intelligent Mechatronics.

[11] Heredia G., Ollero A. Sensor Fault Detection in Small Autonomous Helicopters Using Observer/Kalman Filter Identification. Proceedings of the 2009 IEEE International Conference on Mechatronics.

[12] Isermann R. (2006). Fault-Diagnosis Systems. An Introduction from Fault Detection to Fault Tolerance.

[13] Li X.-J., Yang G.-H. (2012). Dynamic Observer-Based Robust Control and Fault Detection for Linear Systems. IET Control Theory Appl..

[14] Kumar V.E., Jerome J. (2013). Sensor Fault Detection in DC Servo System Using Unknown Input Observer with Structured Residual Generation. J. Electr. Eng..

[15] Yi J., Huang Z., Liu W., Yang Y., Zhang X., Liu J. Actuator Fault Detection Based on Robust Adaptive Observer for CCBII Braking System. Proceedings of the 26th Chinese Control and Decision Conference (2014 CCDC).

[16] Khalid H.M., Khoukhi A., Al-Sunni F.M. Fault Detection and Classification Using Kalman Filter and Genetic Neuro-Fuzzy Systems. Proceedings of the 2011 Annual Meeting of the North American Fuzzy Information Processing Society.

[17] Yang S.K., Liu T.S. (1999). State Estimation for Predictive Maintenance Using Kalman Filter. Reliab. Eng. Syst. Saf..

[18] Alkaya A., Eker İ. (2014). Luenberger Observer-Based Sensor Fault Detection: Online Application to DC Motor. Turk. J. Electr. Eng. Comput. Sci..

[19] Tarantino R., Szigeti F., Colina-Morles E. (2000). Generalized Luenberger Observer-Based Fault-Detection Filter Design: An Industrial Application. Control Eng. Pract..

[20] Schölkopf B., Smola A., Müller K.-R. (1998). Nonlinear Component Analysis as a Kernel Eigenvalue Problem. Neural Comput..

[21] Dong D., McAvoy T.J. (1996). Nonlinear Principal Component Analysis—Based on Principal Curves and Neural Networks. Comput. Chem. Eng..

[22] Fazai R., Taouali O., Harkat M.F., Bouguila N. (2016). A New Fault Detection Method for Nonlinear Process Monitoring. Int. J. Adv. Manuf. Technol..

[23] Harkat M.-F., Mourot G., Ragot J. (2006). An Improved PCA Scheme for Sensor FDI: Application to an Air Quality Monitoring Network. J. Process Control.

[24] Ge Z., Yang C., Song Z. (2009). Improved Kernel PCA-Based Monitoring Approach for Nonlinear Processes. Chem. Eng. Sci..

[25] Vapnik V. (1998). The Support Vector Method of Function Estimation. Nonlinear Modeling: Advanced Black-Box Techniques.

[26] Harkat M.-F., Mourot G., Ragot J. (2009). Multiple Sensor Fault Detection and Isolation of an Air Quality Monitoring Network Using RBF-NLPCA Model. IFAC Proc. Vol..

[27] Jaffel I., Taouali O., Harkat M.F., Messaoud H. (2017). Kernel Principal Component Analysis with Reduced Complexity for Nonlinear Dynamic Process Monitoring. Int. J. Adv. Manuf. Technol..

[28] Jaffel I., Taouali O., Harkat M.F., Messaoud H. (2016). Moving Window KPCA with Reduced Complexity for Nonlinear Dynamic Process Monitoring. ISA Trans..

[29] Kallas M., Mourot G., Maquin D., Ragot J. (2014). Diagnosis of Nonlinear Systems Using Kernel Principal Component Analysis. Proc. J. Phys. Conf. Ser..

[30] Kazor K., Holloway R.W., Cath T.Y., Hering A.S. (2016). Comparison of Linear and Nonlinear Dimension Reduction Techniques for Automated Process Monitoring of a Decentralized Wastewater Treatment Facility. Stoch. Environ. Res. Risk Assess..

[31] Kim K.I., Jung K., Kim H.J. (2002). Face Recognition Using Kernel Principal Component Analysis. IEEE Signal Process. Lett..

[32] Lahdhiri H., Taouali O., Elaissi I., Jaffel I., Harakat M.F., Messaoud H. (2017). A New Fault Detection Index Based on Mahalanobis Distance and Kernel Method. Int. J. Adv. Manuf. Technol..

[33] Lefebvre D., Basile F. (2021). An Approach Based on Timed Petri Nets and Tree Encoding to Implement Search Algorithms for a Class of Scheduling Problems. Inf. Sci..

[34] Lajmi F., Talmoudi A.J., Dhouibi H. (2017). Fault Diagnosis of Uncertain Systems Based on Interval Fuzzy PETRI Net. Stud. Inform. Control.

[35] Fatma L., Ghabi J., Dhouibi H. (2020). Applying Interval Fuzzy Petri Net to Failure Analysis. Int. J. Serv. Sci. Manag. Eng. Technol..

[36] Lee J.-M., Yoo C., Lee I.-B. (2004). Statistical Process Monitoring with Independent Component Analysis. J. Process Control.

[37] Lee J.-M., Yoo C., Choi S.W., Vanrolleghem P.A., Lee I.-B. (2004). Nonlinear Process Monitoring Using Kernel Principal Component Analysis. Chem. Eng. Sci..

[38] Li G., Qin S.J., Zhou D. (2010). Geometric Properties of Partial Least Squares for Process Monitoring. Automatica.

[39] Li H., Zhang D. (2013). Stochastic Representation and Dimension Reduction for Non-Gaussian Random Fields: Review and Reflection. Stoch. Environ. Res. Risk Assess..

[40] Liu X., Kruger U., Littler T., Xie L., Wang S. (2009). Moving Window Kernel PCA for Adaptive Monitoring of Nonlinear Processes. Chemom. Intell. Lab. Syst..

[41] Aizerman A. (1964). Theoretical Foundations of the Potential Function Method in Pattern Recognition Learning. Autom. Remote Control.

[42] Mansouri M., Nounou M., Nounou H., Karim N. (2016). Kernel PCA-Based GLRT for Nonlinear Fault Detection of Chemical Processes. J. Loss Prev. Process Ind..

[43] Mercer J. (1909). XVI. Functions of Positive and Negative Type, and Their Connection the Theory of Integral Equations. Philos. Trans. R. Soc. Lond. Ser. A Contain. Pap. A Math. Phys. Character.

[44] Mika S., Schölkopf B., Smola A., Müller K.-R., Scholz M., Rätsch G. (1998). Kernel PCA and De-Noising in Feature Spaces. Proceedings of the Advances in Neural Information Processing Systems.

[45] Nomikos P., MacGregor J.F. (1995). Multivariate SPC Charts for Monitoring Batch Processes. Technometrics.

[46] Teston A., Piccinini Scolaro T., Kuntz Maykot J., Ghisi E. (2022). Comprehensive Environmental Assessment of Rainwater Harvesting Systems: A Literature Review. Water.

[47] Al-Jayyousi O.R. (2003). Greywater Reuse: Towards Sustainable Water Management. Desalination.

[48] Bahta S.T. (2013). Design and Analyzing of an Off-Grid Hybrid Renewable Energy System to Supply Electricity for Rural Areas: Case Study: Atsbi District, North Ethiopia. Master’s Thesis.

[49] Cheremisinoff P.N. (2019). Handbook of Water and Wastewater Treatment Technology.

[50] Bojan-Dragos C.-A., Szedlak-Stinean A.-I., Precup R.-E., Gurgui L., Hedrea E.-L., Mituletu I.-C. Control Solutions for Vertical Three-Tank Systems. Proceedings of the 2018 IEEE 12th International Symposium on Applied Computational Intelligence and Informatics (SACI).

[51] Peters M.S., Timmerhaus K.D., West R.E. (2003). Plant Design and Economics for Chemical Engineers.

[52] Jolliffe I. (2005). Principal Component Analysis. Encyclopedia of Statistics in Behavioral Science.

[53] Sheriff M.Z., Mansouri M., Karim M.N., Nounou H., Nounou M. (2017). Fault Detection Using Multiscale PCA-Based Moving Window GLRT. J. Process Control.

[54] Taouali O., Elaissi I., Messaoud H. (2015). Dimensionality Reduction of RKHS Model Parameters. ISA Trans..

[55] Tharrault Y., Mourot G., Ragot J., Maquin D. (2008). Fault Detection and Isolation with Robust Principal Component Analysis. Int. J. Appl. Math. Comput. Sci..

[56] Vapnik V.N. (1999). An Overview of Statistical Learning Theory. IEEE Trans. Neural Netw..

[57] Zhang Y., Li S., Teng Y. (2012). Dynamic Processes Monitoring Using Recursive Kernel Principal Component Analysis. Chem. Eng. Sci..

[58] Zhao C., Ma J., Wei Y., Long Y., Ou H., Bao J., Yin J., Liu W., Zhu N., Lu X. (2023). Active Mass Transfer for Printable Electrochemical Sensors with Ultrasonic Stimuli. Mater. Today Commun..

[59] Magni J.-F., Mouyon P. A Generalized Approach to Observers for Fault Diagnosis. Proceedings of the 30th IEEE Conference on Decision and Control.

[60] Zhong M., Yang Y., Sun S., Zhou Y., Postolache O., Ge Y. (2020). Priority-Based Speed Control Strategy for Automated Guided Vehicle Path Planning in Automated Container Terminals. Trans. Inst. Meas. Control.

[61] Wu P., Xiao F., Sha C., Huang H., Sun L. (2020). Trajectory Optimization for UAVs’ Efficient Charging in Wireless Rechargeable Sensor Networks. IEEE Trans. Veh. Technol..

